# Medical Revolution

**Published:** 1911-09-09

**Authors:** 


### MEDICAL REVOLUTION.

The attention paid during the past few months to the relation of the medical profession to the public has possibly been considered by Dr. Macilwaine to provide a suitable occasion for the publication of a book entitled "Medical Revolution."* Dr. Macilwaine has more than once previously been a critic of ways and means, both as regards study and practice, of the medical profession. The path of the critic is always easy; at least so far as the criticism itself is concerned. Yet criticism, if sincere, always deserves consideration and may be stimulating.

If the subject of the relation of the medical man to the public be viewed from a detached standpoint it cannot be denied that his position is incongruous. The medical profession, as a whole, aims at the preservation of public health and the banishment of disease. Yet the individual members of the profession can only exist as members of present-day society by deriving profit from the ravages of disease. This fact might be used as a strong argument for the need of a "medical revolution." Whatever our political opinions may be, we cannot blind our eyes to the fact that forces are at work which will tend more and more to influence the position of the medical man. Some such opinion every thoughtful member of the profession must have formed for himself.

### Medicine and the State.

Dr. Macilwaine, however, deals very briefly with the relation of medicine to the State. The subject is looked at more from within than from without. He criticises in the main the theory and practice of medicine. Some of his statements are undoubtedly startling. He says: "It is no exaggeration to say that the recognition of the constitution as a factor in the causation of disease will necessitate an entire rearrangement of our science and the rewriting of medical text-books." He further adds: "The reader cannot yet fully appreciate the significance of the statement." It is perfectly true that there are remarkable differences in different individuals which must have a physical basis. This difference in physical basis has a bearing upon degenerations, upon resistance to disease, and upon manifestations of disease. We may some day arrive at such a height of scientific accuracy that we may be able to ascertain the chemical difference between one man and another, and not only be able to tell why one man is upset by eating pork while another man thrives upon it, but why one man takes unpleasant circumstances calmly and another loses his temper. If we ever arrive at that pitch of perfection we shall no doubt have to rewrite our text-books. At the present time, however, it seems unnecessary to tell the general public that our conceptions of disease are altogether faulty. The author at considerable length criticises the use of the word "disease" and speaks of "spurious diseases." Nearly all diseases of the skin, for example, are classed under spurious diseases. It apparently is considered that diseases should be classified under causation, and causation only. If our knowledge of medicine were perfect nothing could be more ideal. Since, however, we are still unfortunately in great measure groping in the dark, but are yet obliged to use words to describe collections of facts at present ascertained, we are driven to use some such word as "disease" for these collections of facts. Comments are made later upon the position and usefulness of hospitals, and the suggestion thrown out (which has been made before) that the staffs of the out-patient department of the hospitals should be mainly consultants for cases requiring a second opinion in the district, and that recently qualified men should live for a time in the neighbourhood of hospital, attend the patients at their homes, and use the hospital staff as consultants. If this would not interfere with the work of local practitioners, the method would have its value. A knowledge would certainly be obtained of the poor in their homes and long waiting in the out-patient departments of hospitals avoided.

### An Unorganised Profession.

Another opinion of Dr. Macilwaine is that the profession from the scientific standpoint is unorganised. He suggests that there should be a central body of experienced men who would collect information regarding the causation of disease, which they would "systematise and redistribute regularly to every member of the profession." He has little faith in the pathologist, physician, or specialist. From whom is his experienced central committee to collect information? From those in general practice? Observations everywhere are of value, and observations collected from those in general practice no doubt would play a part in adding to the knowledge of medicine, but it is very doubtful whether there would be sufficient interest shown to give much work to the central body.

The book may be summed up in this: that the author considers the profession to be in great need of paying more attention to the causation of disease. In this we feel him to be wrong. All investigators are working to ascertain the cause of disease. Small facts may sometimes occupy a large place in their minds and hide the view of what is beyond. Yet although the judgment and the vision of investigators may at times be faulty they are trying to see, and those in the general practice of their profession who desire the best interests of their patients are always anxious to learn the views of investigators. It is those in general practice no doubt who, in many instances, are in the most favourable position for proving whether the investigators are right or wrong. While this may be so, it is the investigators who almost invariably lead the way. It falls to the lot of some men to investigate, to others to practise. It is not clear that Dr. Macilwaine has evolved any scheme that will make every medical man both an investigator and one who will practise the art of medicine.

* Published by Messrs. King and Co.

